# Discriminating larvae of two syntopic *Cychramus* species (Coleoptera, Nitidulidae) by means of bar-HRM analysis

**DOI:** 10.1007/s11033-020-05786-9

**Published:** 2020-09-08

**Authors:** Lukas Zangl, Hannes Oberreiter, Herbert Huss, Edith Stabentheiner, Christian Sturmbauer, Stephan Koblmüller

**Affiliations:** 1 Institute of Biology, University of Graz, Universitätsplatz 2, 8010 Graz, Austria; 2 Universalmuseum Joanneum, Studienzentrum Naturkunde, Weinzöttlstraße 16, 8045 Graz, Austria; 3 ÖKOTEAM - Institute for Animal Ecology and Landscape Planning, Bergmanngasse 22, 8010 Graz, Austria; 4 Present Address: 4651 Stadl-Paura, Austria; 5 Institute of Biology, University of Graz, Schubertstraße 51, 8010 Graz, Austria

**Keywords:** Coleoptera, DNA-barcoding, High-resolution melting analysis, Larvae, Mini-barcodes, Sap beetles

## Abstract

Molecular genetic methods are increasingly used to supplement or substitute classical morphology-based species identification. Here, we employ a COI mini-barcode coupled high-resolution melting analysis to quickly, cost-efficiently and reliably determine larvae of two closely related *Cychramus* (Coleoptera, Nitidulidae) species. Euclidean distance comparison (p < 0.01) and a Welch t-test of the melting point temperatures (p < 0.01) provide highly significant statistical evidence for species specific differences in melting and fluorescence curves, thus allowing the assignment of larvae to either of the two species. This protocol serves as a fast, low-cost and low-tech method to discriminate between pairs or groups of closely related species and can be adapted and applied to various ecological research questions.

## Introduction

Many key questions asked today in basic and applied biological research require precise species identifications. Traditionally, species identification is based on morphological characters and depends on the organisms’ internal and/or external structures. However, taxonomic identification based only on morphology can be difficult to virtually impossible or very time consuming when two or more species are morphologically highly similar. Indeed, there is increasing evidence that the diversity of recognized morphospecies does by far not reflect the true species diversity, especially in inconspicuous and small taxa [1, 2]. In addition, certain life stages (i.e., eggs and larvae) or sexes are often morphologically indistinguishable among species [3], complicating inferences about species richness and ecological interactions.

In the last two decades, DNA sequence-based methods facilitated species determination in taxa where due to a shortage of reliable characters, morphological identification is difficult. Especially DNA-barcoding [4], which relies on DNA sequence variation of a short and standardized section of a specific gene or set of genes, has become a widely used tool among biologists. Indeed, this approach proved to be a powerful and invaluable method for discriminating a broad range of organisms [5]. Often even shorter fragments, so-called mini-barcodes, are sufficient for discriminating between closely related species and they are typically used for analyzing samples containing degraded DNA and in metabarcoding approaches to efficiently characterize entire communities [6, 7]. In addition, mini-barcodes can be combined with high resolution melting analysis (bar-HRM), which provides a time- and cost-effective way to discriminate DNA sequences with small, even single, nucleotide differences, thus avoiding the need of sequencing, which is the costliest step in standard DNA barcoding. The method is particularly suited for fast discrimination of a limited number of species [8]. Briefly, following a real-time PCR, the products are denatured by increased temperature and the changes in fluorescence caused by the release of an intercalating dye from the DNA duplex are measured [9]. By comparing the melting curves of unknown samples, i.e. the change in intensity of the fluorescence signal with increasing temperature, with profiles of reliably identified samples, they can be assigned to known species [10, 11].

The sap beetle (Nitidulidae) genus *Cychramus* comprises six valid species, two of which, *C. luteus* and *C. variegatus*, are widely distributed across Eurasia and the only *Cychramus* species reported from Europe. Whereas the beetles are regular flower visitors feeding on pollen [12], with *C. luteus* even reported from bee hives [13], the larvae are found on various fleshy fungi, and are particularly common on representatives of the honey fungus species complex, *Armillaria* spp.[14–16]. Unlike the beetles, which are easy to identify, the larvae are almost indistinguishable based on morphological characteristics, especially at younger stages [17]. Due to a lack of reliable species identification, little is known about the larval presence and population dynamics of each of these two species, or the interaction among them. Because honey fungi are among the most important fungal pathogens of temperate and boreal forests, it is of substantial interest to gain better knowledge which of the mushroom-consuming species is prevalent under certain ecological conditions. To this end we developed a robust bar-HRM assay to rapidly identify larvae of *C. luteus* and *C. variegatus* that will facilitate studying ecological interactions between these two species at the larval stage and fungus-beetle (larvae) interactions, and might be easily adapted to other study systems.

## Materials and methods

### Sampling, species determination and standard COI barcode generation

In total, 38 specimens (25 adult beetles, 13 larvae, Table 1) of the two closely related species *C. luteus* and *C. variegatus* were collected from two localities in Austria. Adult specimens were morphologically identified to species level. Standard-length DNA barcodes (658 bp) were generated for some of these specimens. Initial morphological identification of younger larval stages was omitted due to the scarcity of distinguishing characters. Total genomic DNA was extracted using the DNeasy blood and tissue kit (QIAGEN) following the manufacturer’s instructions. Polymerase chain reaction, enzymatic cleanup and cycle sequencing using C_LepFolF and C_LepFolR [18] followed [19] and [20]. Sequencing products were visualized on a 3130xl capillary sequencer (Applied Biosystems). Sequence editing and alignment was done in MEGA 6.06 [21].

**Table 1:** Information on specimens analyzed in the present study as well as sequences downloaded from online repositories are given

Species	Life stage	ID	Locality	Sampling site	BOLD ID; Acc. No
*C. luteus*	Larva	Cyc1	UA, Gunskirchen	48.1144 N; 13.9433 E	MT881657
	Larva	Cyc2	UA, Gunskirchen	48.1144 N; 13.9433 E	MT881658
	Larva	Cyc3	UA, Gunskirchen	48.1144 N; 13.9433 E	MT881659
	Larva	Cyc4	UA, Gunskirchen	48.1144 N; 13.9433 E	MT881660
	Larva	Cyc5	UA, Gunskirchen	48.1144 N; 13.9433 E	MT881661
	Larva	Cyc6	UA, Gunskirchen	48.1144 N; 13.9433 E	MT881662
	Larva	Cyc7	UA, Gunskirchen	48.1144 N; 13.9433 E	MT881663
	Larva	Cyc8	UA, Gunskirchen	48.1144 N; 13.9433 E	MT881664
	Beetle	Cyc18	UA, Gunskirchen	48.1144 N; 13.9433 E	ANIT002-20; MT890466
	Beetle	Cyc24	UA, Gunskirchen	48.1144 N; 13.9433 E	ANIT008-20; MT890467
	Beetle	Cyc25	UA, Gunskirchen	48.1144 N; 13.9433 E	ANIT009-20; MT890468
	Beetle	Cyc26	UA, Gunskirchen	48.1144 N; 13.9433 E	ANIT010-20; MT890469
	Beetle	Cyc27	UA, Gunskirchen	48.1144 N; 13.9433 E	ANIT011-20; MT890470
	Beetle	Cyc28	UA, Gunskirchen	48.1144 N; 13.9433 E	ANIT012-20; MT884449
	Beetle	Cyc29	UA, Gunskirchen	48.1144 N; 13.9433 E	ANIT013-20; MT884448
	Beetle	Cyc30	UA, Gunskirchen	48.1144 N; 13.9433 E	ANIT014-20; MT884447
	Beetle	Cyc31	UA, Gunskirchen	48.1144 N; 13.9433 E	ANIT015-20; MT890471
	Beetle	Cyc37	ST, Graz	47.0863 N; 15.4616 E	ANIT021-20; MT890472
	Beetle	Cyc38	ST, Graz	47.0863 N; 15.4616 E	ANIT022-20; MT884446
	Beetle	Cyc39	ST, Graz	47.0863 N; 15.4616 E	ANIT023-20; MT890473
	Beetle	Cyc40	ST, Graz	47.0863 N; 15.4616 E	ANIT024-20; MT890474
	Beetle	Cyc41	ST, Graz	47.0863 N; 15.4616 E	ANIT025-20; MT890475
Additional sequences					KJ962607; KJ965813; KJ966832; KJ962410; KJ964017; KJ962846; KM448028; KM446407; KM451876; KM448866; KM448805; KM449494; KM449753; KM452505; KM445184; KM442734; KM446278; KU908905; KU910131; KU916564; KU915694; KU914876; KU910893; KM286278
*C. variegatus*	Larva	Cyc9	UA, Gunskirchen	48.1144 N; 13.9433 E	MT881665
	Larva	Cyc10	UA, Gunskirchen	48.1144 N; 13.9433 E	MT881666
	Larva	Cyc14	UA, Gunskirchen	48.1144 N; 13.9433 E	MT881667
	Beetle	Cyc15	UA, Gunskirchen	48.1144 N; 13.9433 E	ANIT001-20; MT884455
	Larva	Cyc16	UA, Gunskirchen	48.1144 N; 13.9433 E	MT881668
	Larva	Cyc17	UA, Gunskirchen	48.1144 N; 13.9433 E	MT881669
	Beetle	Cyc19	UA, Gunskirchen	48.1144 N; 13.9433 E	ANIT003-20; MT884454
	Beetle	Cyc20	UA, Gunskirchen	48.1144 N; 13.9433 E	ANIT004-20; MT884451
	Beetle	Cyc21	UA, Gunskirchen	48.1144 N; 13.9433 E	ANIT005-20; MT884450
	Beetle	Cyc22	UA, Gunskirchen	48.1144 N; 13.9433 E	ANIT006-20; MT890476
	Beetle	Cyc23	UA, Gunskirchen	48.1144 N; 13.9433 E	ANIT007-20; MT890477
	Beetle	Cyc32	ST, Graz	47.0863 N; 15.4616 E	ANIT016-20; MT884453
	Beetle	Cyc33	ST, Graz	47.0863 N; 15.4616 E	ANIT017-20; MT884452
	Beetle	Cyc34	ST, Graz	47.0863 N; 15.4616 E	ANIT018-20; MT890478
	Beetle	Cyc35	ST, Graz	47.0863 N; 15.4616 E	ANIT019-20; MT890479
	Beetle	Cyc36	ST, Graz	47.0863 N; 15.4616 E	ANIT020-20; MT890480
Additional sequences					KM286238; KJ965586

Acronyms UA and ST denote Upper Austria and Styria respectively

### Primer design for mini-barcodes and validation

**Fig. 1:**
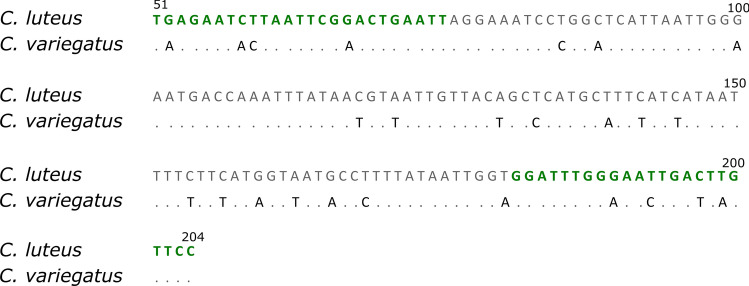
COI sequence alignment of mini-barcode fragments of the two *Cychramus* species. Primer regions are marked in green. (Color figure online)

Additional sequences were downloaded from GenBank (Table 1) to account for geographic genetic variation. A 153 bp fragment spanning from nucleotide position 51 to 204 of the standard barcode fragment containing sufficient nucleotide differences for species discrimination was selected for HRM analysis (Fig. 1). Primers Cyc-HRM-F 5′- TGAGAATCTTAATTCGGACTGAATT and Cyc-HRM-R 5′ GGAACAAGTCAATTTCCAAATCC were designed and their properties (annealing temperature, hairpins, etc.) checked with Primer-BLAST (https://www.ncbi.nlm.nih.gov/tools/primer-blast/). Successful amplification and genetic species determination (including the larvae) by these mini-barcodes was confirmed. Protocols for PCR and cycle sequencing applied as mentioned above, only the PCR annealing temperature (49 °C) differed.

### qPCR and high-resolution melting analysis of COI mini-barcodes

Quantitative real time PCR and subsequent HRM analyses were conducted in a Rotor-Gene 3000 thermal cycler (Corbett Research, Mortlake, New South Wales, Australia). PCR reactions using the Real Time 2 × PCR Master Mix EvaGreen (A & A Biotechnology, Gdynia, Poland) and cycling conditions followed [8], only altering the annealing temperature to 49 °C. Optical measurements at 510 nm were recorded during each extension step. The final extension phase immediately initialized the heating process. Changes in fluorescence were detected during the increase of 0.1 °C increments per second between 60 and 95 °C. qPCR was repeated to obtain a technical replicate. The resulting fluorescence data was visualized using the Rotor-Gene 6.0.27 software.

### Statistical analysis of melting and fluorescence curves

**Fig. 2:**
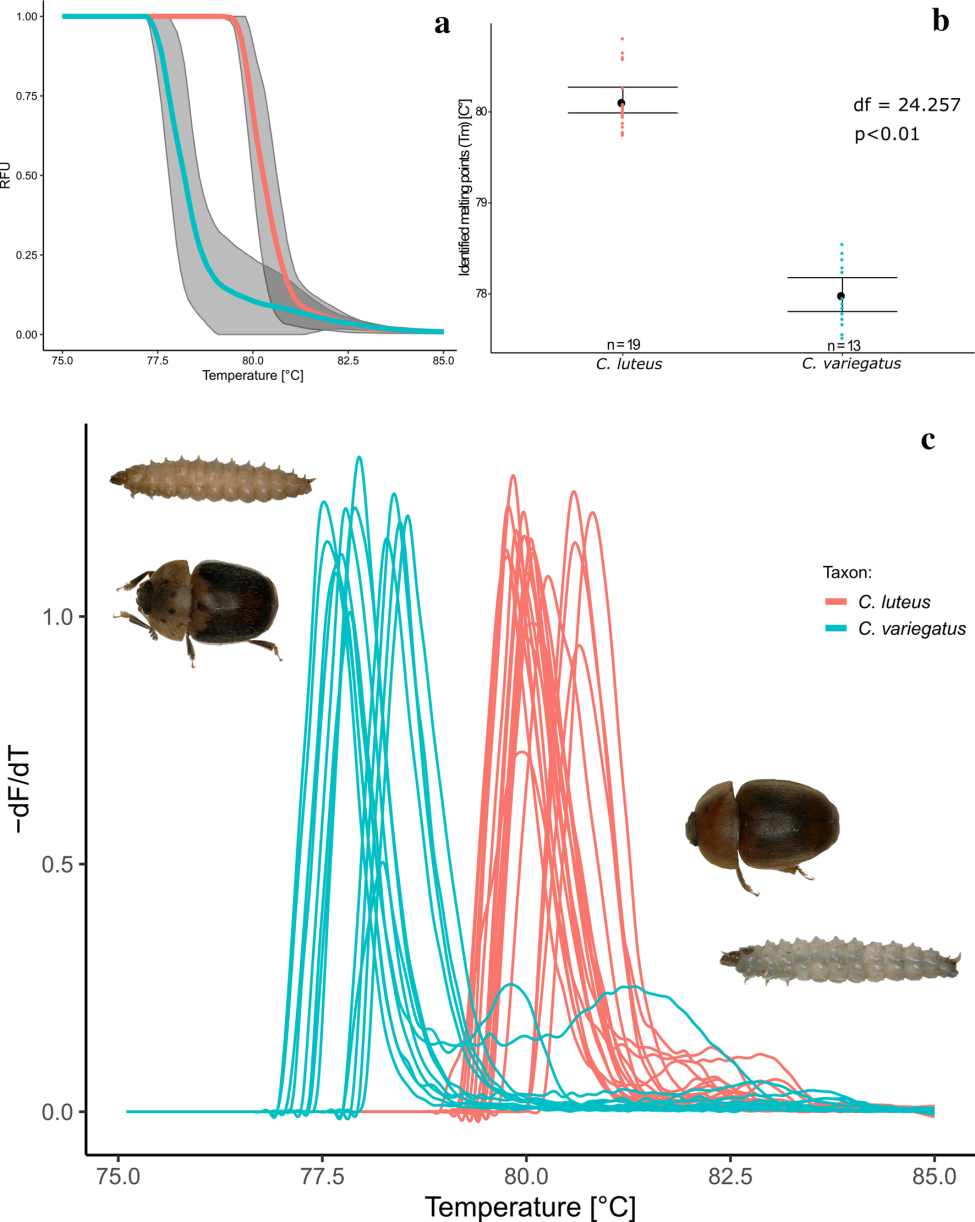
Relative fluorescence curve (**a**), identified melting point comparison (**b**) and melting rate curve (**c**). Species are indicated by blue (*C. variegatus*) and red (*C. lutues*) colors. Colored lines in (a) represent means, grey areas cover standard deviations. Colored dots in (b) mark the distribution of resulting melting points, black dots and error bars represent the means and 95% confidence intervals (bootstrap, BCa 10,000) respectively. (Color figure online)

Statistical analyses were conducted with R version 3.6.3. For reproducibility a docker container was created with Rocker:Tidyverse image 3.6.3 [22, 23]. The R-code and raw relative fluorescence data is publicly available on GitHub and an automatically generated Docker image can be downloaded from Dockerhub. Raw data were normalized at 75 and 85 °C after visual examination of the relative fluorescence decline over time (Fig. 2a). The negative first derivative values (-d(RFU/dT)) from a geometric spline function were used for statistical analysis in the qpcR package. The threshold to identify the melting point (T_m_) was set to 0.2, which resulted in a single peak area for all samples. Distribution analysis was done visually with a Q-Q-plot. The T_m_ from all samples grouped by taxa were compared with a two-sided Welch t-test using a 95% confidence interval and 10,000 bootstrap replicates. Euclidean distance comparison of melt curves followed [24]. p-values below 0.05 were considered significant.

## Results and discussion

The distinction between pairs or groups of sometimes strikingly similar species is often a key element when tackling biological, ecological or conservational research questions [24]. In the recent past, molecular biological methods have increasingly been used to aid in species assignment, albeit often requiring a substantial amount of infrastructure. Although standard genetic methods constantly aim for a reduction in hands-on time and financial efforts, few approaches actually decrease the necessary infrastructure. Here we present a fast, efficient and adaptable way to discriminate morphologically highly similar larvae of two closely related species of sap-feeding beetles of the family Nitidulidae, that will aid in investigating the population dynamics between these two sympatric species, but also the interactions of beetles, fungi and trees [25]. In the present study, we generated 10 new full-length DNA barcodes (MT884446-MT884455) and used them, together with previously published data, as a basis to create primers for a short mini-barcode fragment. High-resolution melting analysis of the mini-barcodes resulted in two clearly separated clusters of melting curves (see Fig. 2b). Subsequent statistical analyses of Euclidean distances (PERMANOVA, df = 1, pseudo-F = 29.6, p < 0.01, 10,000 permutations) and a two-sided Welch t-test (95% CI, df = 24.257, p < 0.01) of the melting point temperatures yielded significant differences in melting and fluorescence curves for *C. luteus* and *C. variegatus* (Fig. 2c), thus allowing for the assignment of the 13 larvae to either of the two species. The significant outcome of these tests indicates that shape, amplitude and melting peak do not just vary by chance [24]. The sensitivity of this method is known to account for single nucleotide differences [8]. The observed consistent differences in melting and fluorescence curves allow for the discrimination of species based on their melting profiles by eye. These results were corroborated by sequencing the short fragments and aligning them to the full-length barcodes. Thus, we conclude that HRM analyses of mini-barcode fragments present an adequate means to reliably differentiate morphologically similar specimens of these closely related species. Our workflow can be easily adapted for many applied and basic research questions whenever time and cost-efficient discrimination of a large number of samples of a limited number of species is necessary. Furthermore, our publicly available R-code can be used for any HRM study to provide statistical corroboration of visual results. Consequently, when short-fragment primers are established, only a qPCR machine and adequate software for visualization is required to facilitate high-sensitivity species discrimination.

## Data Availability

DNA-barcoding data was stored on BOLD, BOLD-IDs and GenBank accession numbers are provided.

## Code availability

All code for the statistical analyses was deposited on GitHub (https://github.com/HannesOberreiter/melt_graz) and archived on zenodo (10.5281/zenodo.38628482) and is publicly available on Dockerhub (https://hub.docker.com/r/hannesoberreiter/melt_graz).
